# Comorbid risks of psychological disorders and gastroesophageal reflux disorder using the national health insurance service—National Sample Cohort: A STROBE-compliant article

**DOI:** 10.1097/MD.0000000000010153

**Published:** 2018-05-04

**Authors:** Ye-Seul Lee, Bo-Hyoung Jang, Seong-Gyu Ko, Younbyoung Chae

**Affiliations:** a Acupuncture and Meridian Science Research Center, College of Korean Medicine; b Department of Preventive Medicine, College of Korean Medicine, Kyung Hee University, Seoul, Republic of Korea.

**Keywords:** comorbidity risks, depression, gastroesophageal reflux disease, National Health Insurance Service, National Sample Cohort, psychological disorders

## Abstract

This study was performed to examine the comorbidity risks between psychological disorders, such as depression, and gastroesophageal reflux disease (GERD) using nationally representative data from a National Sample Cohort of the National Health Insurance Service in Korea.

The National Health Insurance Service—National Sample Cohort (NHIS–NSC) database from 2010 to 2012 was used in this study. GERD patients were defined as those diagnosed with specific tests, with screened medication, and without any other gastrointestinal diseases. Propensity score matching for age, sex, and economic status was applied to form a control cohort. Incidence rate, relative risks, Cox proportional-hazards modeling, and Kaplan–Meier analysis were applied to examine the differences between the GERD and control cohorts with regard to the risk of subsequent psychological disorders.

The results showed that patients in the GERD cohort (n = 9503) had significantly higher risks of psychological disorders than those without GERD (adjusted hazard ratio [HR] 1.25, 95% confidence interval [CI] 1.07–1.47, *P* = .006). Specifically, the risk of depressive disorder was significantly higher for patients in the GERD cohort than in the control cohort (adjusted HR 1.41, 95% CI 1.04–1.91, *P* = .027). Kaplan–Meier analysis showed that the estimated probability of psychological disorders was significantly higher in the GERD cohort compared with the control cohort (log-rank test, *P* = .007).

This study suggested that GERD may be a risk factor for subsequent psychological disorders, specifically, depressive disorder. The results of this study in GERD patients compared with non-GERD patients in Korea suggested that psychological disorders and GERD may be inter-related.

## 1 Introduction

Gastroesophageal reflux disease (GERD) is a chronic condition caused by the reflux of stomach contents, involving heartburn, occasional regurgitation, or retrosternal pain.^[1,2]^ It is one of the most common gastrointestinal (GI) disorders, with prevalence rates of 8.1% to 27.8% in North America, 2.5% to 7.8% in East Asia, and 3.5% to 8.5% in Korea.^[3,4]^ The chronic symptoms of GERD have been shown to negatively influence patients’ psychological well-being, and also functional status and overall work productivity.^[3,5]^ Its primary symptom, heartburn, and its negative impact on well-being were reflected in impairment of validated health-related quality of life measures.^[5–7]^ In addition, healthcare costs related to GERD have been increasing in Asian countries over the past several years.^[8–10]^

Chronic medical conditions, including cardiovascular diseases, which include chest pain as their main symptom, have been shown to be related to psychological disorders.^[11–14]^ Studies regarding chronic GI disorders, such as GERD, have also focused increasingly on its inter-relations with psychological disorders.^[15–19]^ Heartburn, the major symptom of GERD, was shown to be associated with psychological factors; these factors have been investigated as predictors of treatment response in GERD patients.^[15,20,21]^ Studies on GERD have reported associations of GERD with psychological disorders, such as bipolar disorder, sleep disorders, anxiety disorder, and depressive disorders.^[16–19,22,23]^ Other studies suggested the common symptomatic presentation of GERD among patients with psychological disorders.^[24,25]^

Although there have been a number of studies regarding the prevalence and treatment outcomes of GERD patients, there have been no studies from a nationwide or longitudinal perspective using a nationally representative cohort in Korea. This study was performed to examine the relationship between GERD and subsequent risk of psychological disorders through a nationwide, retrospective, 3-year cohort study on the risk of psychological disorders among patients with GERD.

## 2 Methods

### 2.1 Data source

The National Health Insurance Service–National Sample Cohort (NHIS–NSC) is a population-based cohort from 2002 to 2012, established by the National Health Insurance Service (NHIS) in Korea.^[26]^ The NHIS data contain principal and additional diagnoses, hospitalization and outpatient treatment, dates of examinations, medical fees, details of medical services, prescribed medications, hospital codes, and patients’ sex and age, and are categorized based on the examination documented in the claims from medical institutions.^[27]^ The NHIS–NSC data provide personal information, demographics, and medical treatment data based on the NHIS claims from medical institutions from inpatient and outpatient clinic visits for each individual patient.^[26]^ The Korean Classification of Diseases, Sixth revision (KCD-6), was used for coding the diagnoses in the NHIS–NSC database, which is based on the International Classification of Diseases, Tenth Revision, Clinical Modification. To ensure confidentiality, and for ethical considerations, the identification numbers of the individuals in the cohort were encrypted and transformed with a random numeric string. NHIS–NSC 2010, which includes data for 1 million patients, was used for data analysis in this study. Written consent was not obtained from the study participants due to the retrospective nature of the data with encrypted identification of the individuals. A formal waiver of the need for consent was provided for this study by the Institutional Review Board of Kyung Hee University due to its observational methodology using a secondary database.

### 2.2 Study samples

For this research, data from NHIS–NSC 2010 were used to form a retrospective cohort of patients with the principal diagnosis of GERD in their claims data (n = 9053), in which the GERD patient cohort was defined based on previous studies.^[23,28–32]^ In the process of building the cohort, different measures were applied to ensure diagnostic validity. The inclusion criteria included patients above the age of 19 with newly diagnosed GERD according to KCD-6 codes K21.0 (gastroesophageal reflux disease with esophagitis) and K21.9 (gastroesophageal reflux disease without esophagitis), and only the patients with records of endoscopy or 24-hour pH monitoring in 2010 were included to support the diagnoses.

The exclusion criteria included previous medical histories of GERD from 2002 to 2009, histories of any psychological disorder from 2002 to 2009, and before the diagnosis of GERD in 2010, and histories of digestive diseases other than GERD, including inflammatory or ulcerative diseases in the stomach and duodenum, Crohn's disease, inflammatory bowel syndrome, and diseases in the liver or gallbladder.^[28,32]^ Furthermore, patients who had been prescribed antiplatelet medications, including aspirin, along with proton pump inhibitors (PPIs), were excluded, as many antiplatelet and antithrombotic therapies involve combination prescription of PPIs to reduce adverse effects, and the Korean NHIS restricts treatment with oral or intravenous PPIs in patients without a diagnosis of GERD in the claims data.^[29,31,33]^

The control cohort was selected from the NHIS–NSC cohort among patients without GERD and any psychological disorder in 2010, and with no histories of GERD and psychological disorders from 2002 to 2009. For each patient with GERD included in the GERD cohort, age, sex, and economic status were matched to randomly select control patients using the propensity score matching (PSM) method, which is used to balance the confounders and reduce selection bias.^[34–36]^ Each member of the GERD cohort and the matched pair in the control cohort were followed from 2010, and all participants were observed until they were diagnosed with the following psychological diseases: bipolar disorders (KCD-6 codes: F31.0-F31. 9), depressive disorders (KCD-6 codes: F32.0-F32.9, and F33.0-F33.9), mood disorders (KCD-6 codes: F34.0-F34.9, F38.0, F38.1, F38.8, and F39), anxiety disorders (KCD-6 codes: F40.0-F40.9, F41.0-F41.9), obsessive-compulsive disorders (KCD-6 codes: F42.0-F42.9), stress disorders (KCD-6 codes: F43.0-F43.9), somatoform disorders (KCD-6 codes: F45.0-F45.9), and sleep disorders (KCD-6 codes: F51.0-F51.9); or death, until December, 2012.

### 2.3 Comorbidities

The primary clinical outcomes were psychiatrist-diagnosed depressive disorder, bipolar disorder, anxiety disorder, obsessive-compulsive disorder, and sleep disorder. Furthermore, the comorbidities at the date of diagnosis were identified in both GERD and control cohorts. Identified risk factors of GERD include old age, female, lower education and economic status, increase in body mass index (BMI), and tobacco smoking.^[37]^ Although BMI and smoking status were unavailable in the dataset used in this study, we included age, sex, and economic status in the data analysis. In addition, the baseline comorbidities were determined for the patient based on previous studies on comorbid diseases of GERD.^[1,3,30,37–39]^ Baseline comorbidities included in this study included thyroid diseases (KCD-6 codes: E00-E07), diabetes mellitus (KCD-6 codes: E10-E14), obesity (KCD-6 codes: E65-E66), dyslipidemia (KCD-6 codes: E78), chronic obstructive pulmonary disease (COPD) (KCD-6 codes: J44), asthma (KCD-6 codes: J45), and interstitial pulmonary diseases (KCD-6 codes: J84).

### 2.4 Statistical analysis

Demographic characteristics, including age, sex, economic status, and comorbidities of the GERD cohort were compared with the control cohort by chi-square test. The Kaplan–Meier method was applied for cumulative hazard analysis to estimate the probability of the occurrence of psychological disease in the GERD and control cohorts. Univariate and multivariate Cox proportional-hazards models were applied to measure the relative risk of psychological diseases in the study cohort compared with the control cohort to calculate hazard ratios (HRs) and 95% confidence intervals (CIs). The multivariate Cox models included control variables, such as age, sex, and economic status, and common comorbidities, such as thyroid disease, diabetes mellitus, obesity, dyslipidemia, asthma, COPD, and interstitial pulmonary diseases. All statistical analyses were performed using R software (version 3.2.3, “Wooden Christmas-Tree”) and R Studio (Version 0.99.892). The package “MatchIt” was used for the PSM method, and the packages “epitools” and “survival” were used for survival analysis.

## 3 Results

### 3.1 Baseline characteristics of gastroesophageal reflux disease (GERD) and control cohorts

The NHIS–NSC database contained data on 9503 patients with newly diagnosed GERD in 2010. After PSM, 9503 patients were selected as the control cohort, and there were no differences in age, sex, or economic status between the GERD and control cohorts. Table 1 shows the demographic characteristics of GERD and control cohorts. Baseline differences in comorbidities revealed a higher prevalence of asthma among patients in the GERD cohort. Comorbidities of thyroid diseases, diabetes mellitus, and dyslipidemia were higher among patients in the control cohort, and comorbidities of obesity, COPD, and interstitial pulmonary diseases showed no significant differences in prevalence between GERD and the control cohorts (Table 1).

**Table 1:**
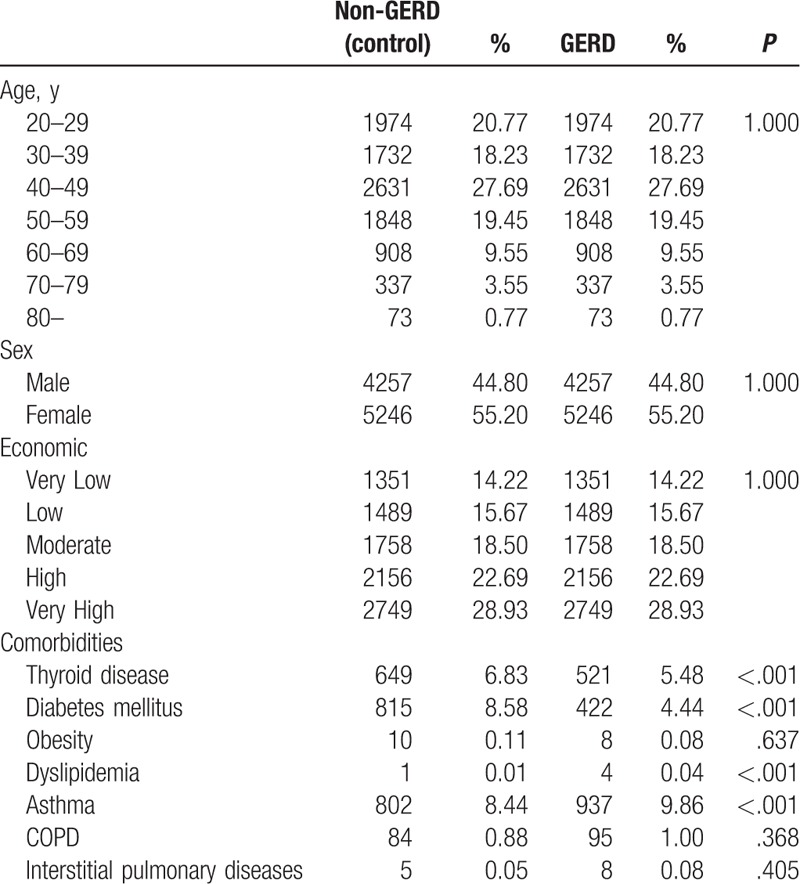
Baseline characteristics of gastroesophageal reflux disease (GERD) and control cohorts.

### 3.2 Incidence of psychological disorders in GERD and control patients

Up to the end of 2012, 367 (3.86%) patients were followed up until the occurrence of psychological disorders in the GERD cohort, and 9105 (95.81%) patients were followed up until the end of the study. In the control cohort, 239 (2.51%) patients were followed up until the occurrence of psychological disorders, and 9235 (97.18%) patients were followed up until the end of the study (*P* < .001). The most common subsequent psychological disorders during the follow-up period were depressive disorder (GERD: 114 patients; control: 68 patients), anxiety disorder (GERD: 102 patients; control: 68 patients), bipolar disorder (GERD: 11 patients; control: 2 patients), and sleep disorder (GERD: 50 patients; control: 34 patients) in both GERD and control cohorts. The incidence rates were significantly higher in the GERD cohort compared with the control cohort for depressive disorder (22.38 vs 17.69 per 1000 person-years, respectively) and anxiety disorder (20.03 vs 17.69 per 1000 person-years, respectively) (depressive disorder: risk ratio [RR] 1.68, *P* < .001; anxiety disorder: RR 1.50, *P* = .009). The incidence rate of bipolar disorder was significantly higher in the GERD cohort compared with the control cohort (2.16 vs 0.52 per 1000 person-years, respectively; RR 5.50, *P* = .013). In the case of sleep disorder, the incidence rate was marginally higher in the GERD cohort compared with the control cohort, but the difference was not statistically significant (9.82 vs 8.84 per 1000 person-years, respectively; RR 1.47, *P* = .080) (Table 2).

**Table 2:**
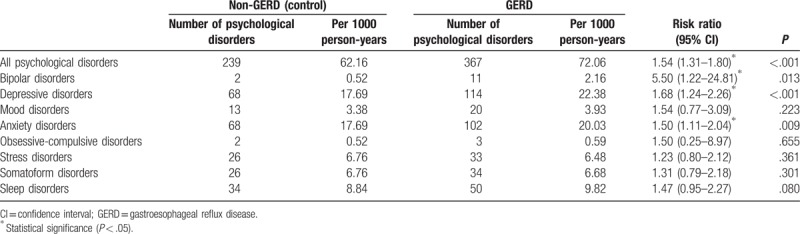
Incidence rates of psychological disorders and their specific categories (bipolar, depressive, anxiety, obsessive-compulsive, stress, somatoform, and sleep disorders) in GERD and control cohorts.

### 3.3 Hazard ratios of psychological disorders in GERD and control patients

**Figure 1:**
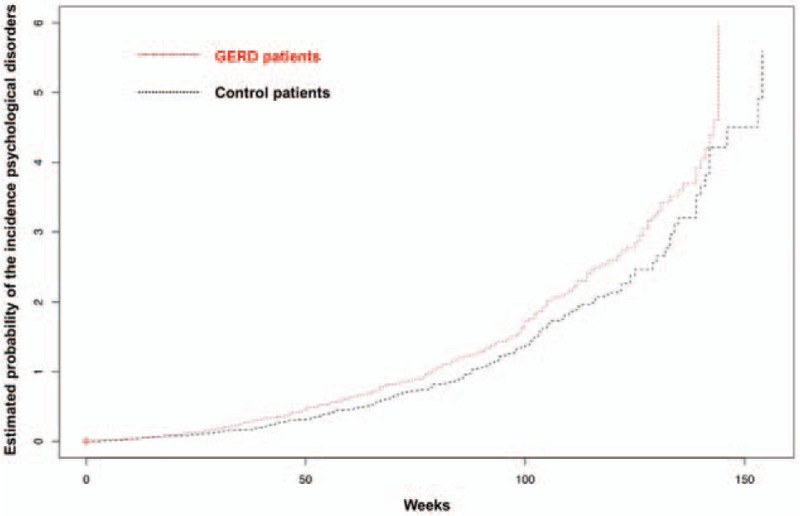
The estimated probability of the incidence of psychological disorders for patients in GERD and control cohorts. The patients in the GERD cohort showed a significantly higher probability of having psychological disorders than those in the control cohort (log-rank test, *P* = .007). X-axis: time to diagnosis of GERD in weeks; y-axis: estimated probability of the incidence of psychological disorders. Red line: GERD cohort patients; black line: control patients. GERD = gastroesophageal reflux disease.

To analyze the HRs of the newly diagnosed psychological disorders for patients in the GERD cohort and control cohort, Cox proportional-hazard regression analysis was conducted for the common psychological disorders (Table 3). The results indicated that, compared with the patients in the control cohort, those in the GERD cohort exhibited a significantly higher risk of subsequent psychological disorders (adjusted HR 1.25, 95% CI 1.07–1.47, *P* = .006). In the Cox hazard ratio model for specific psychological disorders, the HR of depressive disorder (adjusted HR 1.41, 95% CI 1.04–1.91, *P* = .027) in the GERD cohort was significantly higher than that in the control cohort. Figure 1 shows the Kaplan–Meier curves of cumulative hazards of psychological disorders between GERD and control cohorts between 2010 and 2012. Kaplan–Meier analysis showed that the estimated probability of psychological disorders was significantly higher in patients in the GERD cohort compared to those in the control cohort (log rank test, *P* = .007).

**Table 3:**
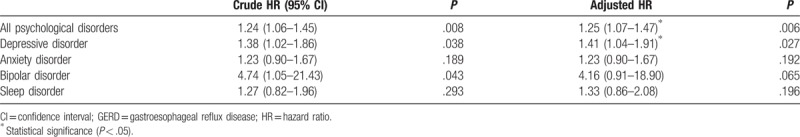
Hazard ratios of time until psychological disorders appear in GERD and control cohort patients.

## 4 Discussion

The results of the present study indicated that GERD was significantly associated with increased sequential risk of psychological disorders based on a nationwide representative cohort database. The most common psychological disorders among patients with GERD and those without GERD in Korea were depressive disorder, anxiety disorder, bipolar disorder, and sleep disorder. Based on the PSM method, the selection bias between GERD and control cohort was minimized, and it was possible to estimate the effect of GERD on the incidence of psychological disorders. Among these categories of psychological disorders, depressive disorder showed a significantly higher subsequent incidence after the diagnosis of GERD compared to patients without GERD.

This study suggested that GERD may be a risk factor for subsequent psychological disorders, specifically depressive disorder. A number of studies demonstrated such relationships between GERD and psychological disorders, such as bipolar disorder, sleep disorders, anxiety disorder, and depressive disorders.^[16–19,22,23]^ Heartburn, the major symptom of GERD, has been shown to be associated with psychological factors, and these factors have been investigated as predictors of treatment response in GERD patients.^[15,20,21]^ Anxiety and depression develop after GERD, which, in turn, cause increased sensitivity to the reflux symptoms.^[40]^ Conversely, symptomatic presentation of GERD and severity of reflux were shown to be common among patients with psychological disorders.^[24,25,40]^ According to previous studies, this relationship between GERD and psychological symptoms may be attributable to the mechanism of chronic inflammation.^[41–44]^ Inflammatory cytokines and chemokines, including interleukin (IL)-6, IL-8, IL-1β, tumor necrosis factor-α, platelet activating factor, and reactive oxygen species, are present in the esophageal mucosa of patients with GERD.^[41,43]^ Other studies indicated associations between psychological disorders, such as depressive and anxiety disorders, and chronic and mild inflammation in the peripheral blood and in the brain.^[42,44,45]^

Our findings were consistent with previous studies in which the prevalence rates of asthma were higher among patients with GERD.^[1,9,30,38,43]^ Previous studies have demonstrated that altered respiratory physiology in asthma patients may predispose them toward GERD, due to negative pleural pressure from the respiratory obstruction that is causing the reflux.^[1,38]^ On the contrary, the comorbidities of other diseases in our study, such as diabetes mellitus, dyslipidemia, and chronic pulmonary diseases, were inconsistent with previous studies showing higher prevalence rates of such diseases in GERD patients.^[1,3,23,30,37–39]^ This may have been due to the characteristics of the study itself. Our findings must be replicated in future studies. In addition, we excluded patients receiving antiplatelet and antithrombotic therapies to ensure that only GERD patients were included in our study, which may have limited the prevalence of other chronic diseases in the baseline comorbidities. However, the number of cases and the controls, and also the duration of the study, seemed to provide sufficient evidence for this finding, and our results suggest that the characteristics of baseline comorbidities in GERD patients from Korea may be different from those in other countries.

The incidence of psychological disorders in our study was 3.86% from 2010 to 2012, whereas the prevalence rates of psychological disorders, including depressive, anxiety, and sleep disorders, were approximately 20% in previous studies.^[19,24]^ This difference may be explained first through the difference between the incidence and prevalence rates—this study included the incidence rates of psychological disorders, whereas other studies have analyzed the prevalence rates of psychological disorders among GERD patients. Second, while previous studies employed rating scales for evaluation of depressive and anxiety disorders, our study included the outcome as diagnosis made by psychiatrists in the claims data. This difference in definition of the outcome may have led to a decrease in diagnosis numbers in our study. On the contrary, a recent study in Taiwan indicated incidence rates of psychological disorders among GERD patients of 1.18% to 1.99%, which were lower than those in our study.

Our study revealed a relatively lower prevalence of comorbid diseases among GERD patients and relatively higher outcomes of psychological disorders compared with previous studies in other countries.^[19,24]^ One explanation may lie in the characteristics of the psychosomatic disorders among Koreans. A number of studies have reported distinct somatic symptoms of various psychological disorders in East Asia.^[46]^ The presence of painful physical symptoms in depressive disorder was associated with poor outcomes in East Asia, and also in non-Asian populations.^[47]^ Somatization refers to the tendency to emphasize somatic symptoms when experiencing a psychiatric disturbance, and this tendency has been widely reported in patients from Korea and other East Asian countries.^[46–48]^ A previous study in Korea suggested that esophageal symptoms of patients with abnormal esophageal motility may be related to the underlying psychological abnormalities, independent of manometric abnormalities, implicating the esophageal symptoms as a subconscious presentation of psychosomatic symptoms.^[49]^ Another study indicated that somatization, the symptoms of which include heartburn or chest pain, found in Korean depression patients, was significantly correlated with resilience during the peak of depression, which involves exhibiting physical conditions while subliminally trying to suppress their psychological disturbances, and this may be related to cultural factors.^[50]^

While the treatments of GERD include diet cautions by avoiding certain types of food and changing diet patterns which can worsen the symptoms, and medications such as antacids, H2 blockers, and PPIs,^[7,29,31,43]^ our study also implies the role of medications for psychological disorders to treat GERD symptoms. Previous studies have already mentioned the role of selective serotonin reuptake inhibitors in influencing esophageal perception.^[20,51]^ Furthermore, treatments to relieve psychosomatic symptoms including heartburn^[11,20,23]^ may be of help for those patients who have both GERD and psychological disorders such as depressive disorder.

While the relationships between esophageal symptoms, including heartburn and chest pain, with psychological disorders, were discussed in depth in previous studies, some psychological disorders, including depression and anxiety disorders, indicate chest pain as a somatic symptom of psychological distress.^[5,24]^ Furthermore, the cultural disorder in Korea known as “*Hwa-byung*,” is a psychological disorder, the main symptoms of which are somatic symptoms of chest pain and heartburn.^[52,53]^ It has been reported in previous studies that while this is recognized as a cultural disorder in Korea, some doctors diagnose and equate *Hwa-byung* with depression.^[53]^ Previous studies also suggested the possible interpretation that *Hwa-byung* and the somatic symptoms of heartburn and chest pain may be a culturally patterned way of expression for Koreans experiencing major depression and related conditions.^[54–56]^ Based on these studies, the higher incidence of psychological disorders in the GERD cohort compared with the control cohort, despite similar prevalence rates of baseline comorbidities, may be due to a form of culturally patterned expression of psychological distress, similarly mentioned in both depression and *Hwa-byung*.

This is the first 3-year longitudinal study in Korea investigating the comorbidity risk of psychological disorders among GERD patients with a nationwide sample database. Our study benefited from the large sample size and diagnoses of GERD through tests, such as endoscopy or 24-hour pH monitoring, and also diagnoses of psychological disorders by psychiatric specialists. Furthermore, our study avoided the possibly inaccurate diagnosis of GERD by analyzing the medical prescriptions, as patients may require supplementary prescription of GERD medications in addition to their main illness. In addition, the study design avoided selection bias in the study population by using a nationally representative cohort, as universal health coverage in Korea ensures that all Koreans are enlisted in the NHIS database, and their medical conditions and healthcare utilization are followed up until death of the individual.^[26]^

Several limitations to our findings must be taken into consideration. First, as we excluded patients receiving antiplatelet and antithrombotic therapies to ensure diagnostic validity of GERD, the baseline characteristics of cardiovascular and cerebrovascular disease prevalence were unavailable. Second, the NHIS–NSC database does not include detailed information on patients, such as smoking status, alcohol consumption, BMI, diet, and family history of psychological disorders. Therefore, we could not control for these factors, which may potentially confound the results.

In conclusion, the results of this study suggested that GERD increases the risk of psychological disorders, such as depression. The higher incidence of psychological disorders among GERD patients compared with non-GERD patients in Korea may be due to psychological factors. Further clinical studies on the relationship between GERD and psychological disorders are required.

## Author contributions

**Project administration:** Y. Chae.

**Supervision:** S-G. Ko.

**Writing – original draft:** Y-S. Lee.

**Writing – review & editing:** B-H. Jang.

## References

[1] Harding SM Richter JE Guzzo MR Asthma and gastroesophageal reflux: acid suppressive therapy improves asthma outcome. Am J Med 1996;100:395–405. 8610725 10.1016/S0002-9343(97)89514-9

[2] Mikami DJ Murayama KM Physiology and pathogenesis of gastroesophageal reflux disease. Surg Clin North Am 2015;95:515–25. 25965127 10.1016/j.suc.2015.02.006

[3] El-Serag HB Sweet S Winchester CC Update on the epidemiology of gastro-oesophageal reflux disease: a systematic review. Gut 2014;63:871–80. 23853213 10.1136/gutjnl-2012-304269 PMC4046948

[4] Cho YS Choi MG Jeong JJ Prevalence and clinical spectrum of gastroesophageal reflux: a population-based study in Asan-si, Korea. Am J Gastroenterol 2005;100:747–53. 15784014 10.1111/j.1572-0241.2005.41245.x

[5] Denver P Donnelly M Murray LJ Psychosocial factors and their association with reflux oesophagitis, Barrett's oesophagus and oesophageal adenocarcinoma. World J Gastroenterol 2013;19:1770–7. 23555165 10.3748/wjg.v19.i11.1770 PMC3607753

[6] Dimenas E Carlsson G Glise H Relevance of norm values as part of the documentation of quality of life instruments for use in upper gastrointestinal disease. Scand J Gastroenterol Suppl 1996;221:8–13. 9110389 10.3109/00365529609095544

[7] Revicki DA Wood M Maton PN The impact of gastroesophageal reflux disease on health-related quality of life. Am J Med 1998;104:252–8. 9552088 10.1016/s0002-9343(97)00354-9

[8] Sandler RS Everhart JE Donowitz M The burden of selected digestive diseases in the United States. Gastroenterology 2002;122:1500–11. 11984534 10.1053/gast.2002.32978

[9] Jung HK Epidemiology of gastroesophageal reflux disease in Asia: a systematic review. J Neurogastroenterol Motil 2011;17:14–27. 21369488 10.5056/jnm.2011.17.1.14 PMC3042214

[10] Lu CL Lang HC Chang FY Social and medical impact, sleep quality and the pharmaceutical costs of heartburn in Taiwan. Aliment Pharmacol Ther 2005;22:739–47. 16197495 10.1111/j.1365-2036.2005.02664.x

[11] White KS Assessment and treatment of psychological causes of chest pain. Med Clin North Am 2010;94:291–318. 20380957 10.1016/j.mcna.2010.01.005

[12] Strodl E Kenardy J A history of heart interventions moderates the relationship between psychological variables and the presence of chest pain in older women with self-reported coronary heart disease. Br J Health Psychol 2013;18:687–706. 23217000 10.1111/bjhp.12011

[13] Kisely SR The relationship between admission to hospital with chest pain and psychiatric disorder. Aust N Z J Psychiatry 1998;32:172–9. 9588295 10.3109/00048679809062726

[14] Wheeler A Schrader G Tucker G Prevalence of depression in patients with chest pain and non-obstructive coronary artery disease. Am J Cardiol 2013;112:656–9. 23711812 10.1016/j.amjcard.2013.04.042

[15] Naliboff BD Mayer M Fass R The effect of life stress on symptoms of heartburn. Psychosom Med 2004;66:426–34. 15184707 10.1097/01.psy.0000124756.37520.84

[16] Shapiro M Simantov R Yair M Comparison of central and intraesophageal factors between gastroesophageal reflux disease (GERD) patients and those with GERD-related noncardiac chest pain. Dis Esophagus 2012;25:702–8. 22309285 10.1111/j.1442-2050.2011.01317.x

[17] Lin WS Hu LY Liu CJ Gastroesophageal reflux disease and risk for bipolar disorder: a nationwide population-based study. PLoS One 2014;9:e107694. 25255080 10.1371/journal.pone.0107694 PMC4177834

[18] Kim JY Kim N Seo PJ Association of sleep dysfunction and emotional status with gastroesophageal reflux disease in Korea. J Neurogastroenterol Motil 2013;19:344–54. 23875102 10.5056/jnm.2013.19.3.344 PMC3714413

[19] Mody R Bolge SC Kannan H Effects of gastroesophageal reflux disease on sleep and outcomes. Clin Gastroenterol Hepatol 2009;7:953–9. 19375520 10.1016/j.cgh.2009.04.005

[20] Wiklund I Carlsson R Carlsson J Psychological factors as a predictor of treatment response in patients with heartburn: a pooled analysis of clinical trials. Scand J Gastroenterol 2006;41:288–93. 16497615 10.1080/00365520500292970

[21] Johnston BT Stress and heartburn. J Psychosom Res 2005;59:425–6. 16310025 10.1016/j.jpsychores.2005.05.011

[22] Jung HK Choung RS Talley NJ Gastroesophageal reflux disease and sleep disorders: evidence for a causal link and therapeutic implications. J Neurogastroenterol Motil 2010;16:22–9. 20535322 10.5056/jnm.2010.16.1.22 PMC2879818

[23] You ZH Perng CL Hu LY Risk of psychiatric disorders following gastroesophageal reflux disease: a nationwide population-based cohort study. Eur J Intern Med 2015;26:534–9. 26021838 10.1016/j.ejim.2015.05.005

[24] Jansson C Nordenstedt H Wallander MA Severe gastro-oesophageal reflux symptoms in relation to anxiety, depression and coping in a population-based study. Aliment Pharmacol Ther 2007;26:683–91. 17697202 10.1111/j.1365-2036.2007.03411.x

[25] Ju G Yoon IY Lee SD Relationships between sleep disturbances and gastroesophageal reflux disease in Asian sleep clinic referrals. J Psychosom Res 2013;75:551–5. 24290045 10.1016/j.jpsychores.2013.10.004

[26] Lee J Lee JS Park SH Cohort profile: The National Health Insurance Service-National Sample Cohort (NHIS-NSC), South Korea. Int J Epidemiol 2017;46:e15. 26822938 10.1093/ije/dyv319

[27] Jee SH Park JW Lee SY Stroke risk prediction model: a risk profile from the Korean study. Atherosclerosis 2008;197:318–25. 17586511 10.1016/j.atherosclerosis.2007.05.014

[28] de Bortoli N Martinucci I Bellini M Overlap of functional heartburn and gastroesophageal reflux disease with irritable bowel syndrome. World J Gastroenterol 2013;19:5787–97. 24124323 10.3748/wjg.v19.i35.5787 PMC3793133

[29] Heidelbaugh JJ Kim AH Chang R Overutilization of proton-pump inhibitors: what the clinician needs to know. Therap Adv Gastroenterol 2012;5:219–32. 10.1177/1756283X12437358 PMC3388523 22778788

[30] Lee CM Lee DH Ahn BK Protective effect of proton pump inhibitor for survival in patients with gastroesophageal reflux disease and idiopathic pulmonary fibrosis. J Neurogastroenterol Motil 2016;22:444–51. 26932897 10.5056/jnm15192 PMC4930299

[31] Ryu HY Kim JW Kim HS Second-look endoscopy is not associated with better clinical outcomes after gastric endoscopic submucosal dissection: a prospective, randomized, clinical trial analyzed on an as-treated basis. Gastrointest Endosc 2013;78:285–94. 23531425 10.1016/j.gie.2013.02.008

[32] Yarandi SS Nasseri-Moghaddam S Mostajabi P Overlapping gastroesophageal reflux disease and irritable bowel syndrome: increased dysfunctional symptoms. World J Gastroenterol 2010;16:1232–8. 20222167 10.3748/wjg.v16.i9.1232 PMC2839176

[33] Lee WH Chu CY Hsu PC Comparison of antiplatelet and antithrombotic therapy for secondary prevention of ischemic stroke in patients with peripheral artery disease: population-based follow-up study in Taiwan. Circ J 2013;77:1046–52. 23269006 10.1253/circj.cj-12-1122

[34] Lu L Gong X Zeng J Health status and migration: a propensity score matching with difference-in-difference regression approach. Lancet 2016;388 suppl 1:S60.

[35] Cluver L Boyes M Orkin M Child-focused state cash transfers and adolescent risk of HIV infection in South Africa: a propensity-score-matched case-control study. Lancet Glob Health 2013;1:e362–70. 25104601 10.1016/S2214-109X(13)70115-3

[36] Juo YY Hyder O Haider AH Is minimally invasive colon resection better than traditional approaches?: First comprehensive national examination with propensity score matching. JAMA Surg 2014;149:177–84. 24352653 10.1001/jamasurg.2013.3660 PMC4036435

[37] Hallan A Bomme M Hveem K Risk factors on the development of new-onset gastroesophageal reflux symptoms. A population-based prospective cohort study: the HUNT study. Am J Gastroenterol 2015;110:393–400. [quiz 401]. 25665934 10.1038/ajg.2015.18

[38] Ruigomez A Rodriguez LA Wallander MA Gastroesophageal reflux disease and asthma: a longitudinal study in UK general practice. Chest 2005;128:85–93. 16002920 10.1378/chest.128.1.85

[39] Chen CH Lin CL Kao CH Association between gastroesophageal reflux disease and coronary heart disease: a nationwide population-based analysis. Medicine (Baltimore) 2016;95:e4089. 27399102 10.1097/MD.0000000000004089 PMC5058831

[40] Oh JH Kim TS Choi MG Relationship between psychological factors and quality of life in subtypes of gastroesophageal reflux disease. Gut Liver 2009;3:259–65. 20431758 10.5009/gnl.2009.3.4.259 PMC2852738

[41] Altomare A Guarino MP Cocca S Gastroesophageal reflux disease: update on inflammation and symptom perception. World J Gastroenterol 2013;19:6523–8. 24151376 10.3748/wjg.v19.i39.6523 PMC3801363

[42] Marques AH Cizza G Sternberg E [Brain-immune interactions and implications in psychiatric disorders]. Rev Bras Psiquiatr 2007;29 suppl 1:S27–32. 17546344 10.1590/s1516-44462007000500006

[43] Rieder F Biancani P Harnett K Inflammatory mediators in gastroesophageal reflux disease: impact on esophageal motility, fibrosis, and carcinogenesis. Am J Physiol Gastrointest Liver Physiol 2010;298:G571–81. 20299604 10.1152/ajpgi.00454.2009 PMC2867418

[44] Salim S Chugh G Asghar M Inflammation in anxiety. Adv Protein Chem Struct Biol 2012;88:1–25. 22814704 10.1016/B978-0-12-398314-5.00001-5

[45] Schiepers OJ Wichers MC Maes M Cytokines and major depression. Prog Neuropsychopharmacol Biol Psychiatry 2005;29:201–17. 15694227 10.1016/j.pnpbp.2004.11.003

[46] Zhou X Min S Sun J Extending a structural model of somatization to South Koreans: cultural values, somatization tendency, and the presentation of depressive symptoms. J Affect Disord 2015;176:151–4. 25721611 10.1016/j.jad.2015.01.040

[47] Ang QQ Wing YK He Y Association between painful physical symptoms and clinical outcomes in East Asian patients with major depressive disorder: a 3-month prospective observational study. Int J Clin Pract 2009;63:1041–9. 19570122 10.1111/j.1742-1241.2009.02107.x

[48] Saint Arnault D Kim O Is there an Asian idiom of distress? Somatic symptoms in female Japanese and Korean students. Arch Psychiatr Nurs 2008;22:27–38. 18207054 10.1016/j.apnu.2007.10.003 PMC2239242

[49] Song CW Lee SJ Jeen YT Inconsistent association of esophageal symptoms, psychometric abnormalities and dysmotility. Am J Gastroenterol 2001;96:2312–6. 11513167 10.1111/j.1572-0241.2001.04035.x

[50] Um YH Huh HJ Kim SY Possible cultural effects on the increments of somatic symptoms in subjectively resilient depressed patients. Asia Pac Psychiatry 2014;6:259–66. 25103955 10.1111/appy.12143

[51] Viazis N Karamanolis G Vienna E Selective-serotonin reuptake inhibitors for the treatment of hypersensitive esophagus. Therap Adv Gastroenterol 2011;4:295–300. 10.1177/1756283X11409279 PMC3165206 21922028

[52] Min SK Suh SY The anger syndrome hwa-byung and its comorbidity. J Affect Disord 2010;124:211–4. 19880191 10.1016/j.jad.2009.10.011

[53] Suh S Stories to be told: Korean doctors between hwa-byung (fire-illness) and depression, 1970-2011. Cult Med Psychiatry 2013;37:81–104. 23229388 10.1007/s11013-012-9291-x PMC3585958

[54] Lin KM Lau JK Yamamoto J Hwa-byung. A community study of Korean Americans. J Nerv Ment Dis 1992;180:386–91. 1593273

[55] Choi H Stafford L Meininger JC Psychometric properties of the DSM scale for depression (DSD) with Korean-American youths. Issues Ment Health Nurs 2002;23:735–56. 12523952 10.1080/01612840260433631

[56] Lee J Wachholtz A Choi KH A review of the korean cultural syndrome Hwa-Byung: suggestions for theory and intervention. Asia Taepyongyang Sangdam Yongu 2014;4:49. 25408922 10.18401/2014.4.1.4 PMC4232959

